# TGF‐β Initiates β‐Catenin‐Mediated CTGF Secretory Pathway in Old Bovine Nucleus Pulposus Cells: A Potential Mechanism for Intervertebral Disc Degeneration

**DOI:** 10.1002/jbm4.10069

**Published:** 2018-07-10

**Authors:** Qiuqian Wu, Chun Mathers, Ernest W Wang, Sen Sheng, David Wenkert, Jason H Huang

**Affiliations:** ^1^ Department of Neurosurgery Institute for Translational Medicine Baylor Scott & White Health Temple TX USA; ^2^ Division of Endocrinology Baylor Scott & White Health Temple TX USA; ^3^ Department of Neurosurgery Baylor Scott & White Health Temple TX USA

**Keywords:** TGF‐β, β‐CATENIN, CTGF/CCN2, SMURF2, INTERVERTEBRAL DISC DEGENERATION

## Abstract

We have recently demonstrated that overexpression of Smurf2 under the control of type II collagen alpha 1 (*Col2a1*) promoter induces an intervertebral disc degeneration phenotype in *Col2a1‐Smurf2* transgenic mice. The chondrocyte‐like cells that express type II collagen and Smurf2 in the transgenic mouse discs are prone to degenerate. However, how the chondrocyte‐like cells contribute to disc degeneration is not known. Here, we utilized primary old bovine nucleus pulposus (NP) cells as substitutes for the chondrocyte‐like cells in *Col2a1‐Smurf2* transgenic mouse discs to identify mechanism. We found that 35% of the cells were senescent; TGF‐β treatment of the cells induced a rapid moderate accumulation of β‐catenin, which interacted with connective tissue growth factor (CTGF/CCN2) in the cytoplasm and recruited it to the membrane for secretion. The TGF‐β‐initiated β‐catenin‐mediated CTGF secretory cascade did not occur in primary young bovine NP cells; however, when Smurf2 was overexpressed in young bovine NP cells, the cells became senescent and allowed this cascade to occur. These results suggest that Smurf2‐induced disc degeneration in *Col2a1‐Smurf2* transgenic mice occurs through activation of CTGF secretory pathway in senescent disc cells. © 2018 The Authors *JBMR Plus* published by Wiley Periodicals, Inc. on behalf of American Society for Bone and Mineral Research.

## Introduction

Connective tissue growth factor (CTGF, CCN2) is a member of the CYR/CTGF/NOV (CCN) family.^1^ CTGF is an integrin‐binding, extracellular matrix (ECM)‐associated, and cysteine‐rich peptide that promotes cellular adhesion, migration, differentiation, proliferation, and ECM synthesis during chondrogenesis, wound healing, and fibrotic diseases.^2^, ^3^, ^4^, ^5^ CTGF was initially isolated from the conditioned media of human endothelial cells and identified as a mitogenic and chemotactic connective tissue growth factor with a molecular weight of 36 to 38 kDa.^6^ Unlike the endothelial cells that constitutively express CTGF and secrete it into culture media, adult human dermal fibroblasts normally do not express CTGF; although it is strongly induced by exogenous TGF‐β,^7^ CTGF is barely detectable in conditioned media because it associates with the ECM after secretion.^8^ However, the expression levels of CTGF in chondrocytes and the ratio of CTGF associated with ECM to that secreted into media vary with chondrocyte maturation stages. For example, hypertrophic chondrocytes express higher CTGF levels than proliferating chondrocytes;^9^ CTGF secreted by cultured confluent chondrocytes is mainly associated with the ECM, whereas CTGF secreted by the cells at the growing phase is significantly released into media. Moreover, growing chondrocytes secrete CTGF immediately after synthesis; however, confluent chondrocytes retain the synthesized CTGF molecules within the cells for a certain number of hours and secrete them gradually.^10^ It is obvious that the CTGF expression pattern, secretion rate, and diffusion distance within the ECM are dependent on cell type, cell context, and matrix components. However, it is unknown how CTGF secretion is controlled and regulated.

Wound healing is a complex process that includes inflammation, granulation tissue formation, and tissue remodeling phases.^11^ TGF‐β released during the inflammatory phase attracts fibroblasts to the injury site and activates fibroblast proliferation and CTGF expression.^7^, ^12^ CTGF, along with TGF‐β, attracts more fibroblasts and stimulates proliferation and ECM synthesis.^4^, ^7^ Once an abundant connective tissue has been deposited in the wound, TGF‐β and CTGF expression are decreased to basal levels, fibroblasts stop producing ECM, and the fibroblast‐rich granulation tissue is replaced by an acellular scar.^7^, ^11^ However, if this process is not appropriately terminated, fibrosis can occur. For example, in scleroderma patients, CTGF is persistently highly expressed in fibroblasts in affected fibrotic lesions and constitutively activates ECM synthesis, leading to uncontrolled connective tissue deposition and progressive scarring and fibrosis.^3^

The intervertebral disc is a specialized type of connective tissue but possesses a limited ability to repair when it is disrupted. A disc consists of an annulus fibrosus (AF) ring, a nucleus pulposus (NP) core, and two cartilaginous superior and inferior endplates. The outer AF is made up of highly ordered collagen lamellae in which type I collagen fibers are aligned with elongated fibroblasts.^13^ Relative to the outer AF, the inner AF is more like cartilage, containing spherical chondrocyte‐like cells (CLCs) and greater amount of type II collagen and proteoglycans.^13^ The central NP, a gelatinous tissue, is predominantly composed of proteoglycans produced by large notochordal cells and CLCs.^13^ The AF, the NP, and the endplates are interconnected to form the most important part of the motion segment of the spine, allowing the disc to function as a shock absorber and to resist tensile and torsional forces. Disc degeneration is an age‐related process and starts early in life in some species including humans and cattle, which is because the notochordal cells diminish rapidly after birth and are gradually replaced by much smaller CLCs,^14^ contributing to the NP becoming dehydrated and cartilage‐like by adulthood.^13^, ^15^ In the early stage of disc degeneration, clefts/tears occur in the NP and the inner AF, and CLCs in the inner AF proliferate (cloning) and produce matrix in the vicinity of the structural defects.^13^, ^16^ However, the regenerated tissue cannot withstand the daily loading of the spine, leading to structural defect progression. As disc degeneration advances, clefts/tears extend into the outer AF; fibroblasts in the outer AF differentiate into CLCs and deposit matrix; CLCs in the inner AF and endplates form large clones and migrate into the NP.^13^, ^16^, ^17^ In the late stage of disc degeneration, collagen content and cross‐linking increase throughout the disc; the distinction between the anatomic regions is no longer possible and the entire disc becomes fibrotic and scar‐like.^16^, ^17^ Although the process of disc degeneration shares certain similarities with that of wound healing and scleroderma, the underlying mechanisms are largely unknown, which is mainly for the following two reasons. First, the heterogeneity of the disc cells makes it difficult to generate animal models by genetically targeting distinct cell types simultaneously. Second, it is impossible to obtain enough of a subset of genetically modified disc cells for further biochemical studies beyond disc phenotype in mice.^18^, ^19^, ^20^, ^21^

Smad ubiquitin regulatory factor (Smurf) 2, a E3 ubiquitin ligase, was highly detected in human degenerated articular cartilage, and overexpression of Smurf2 under the control of type II collagen alpha 1 promoter (*Col2a1*) induces osteoarthritis in *Col2a1‐Smurf2* transgenic mice.^22^ We have recently shown that *Col2a1‐Smurf2* transgenic mice also exhibit accelerated age‐related intervertebral disc degeneration.^23^ During development of the disc degeneration in these transgenic mice, many phenotypic changes such as fibroblast‐to‐CLC differentiation, CLC cloning, migration, and fibrosis were similar to those occurring in humans; CTGF expression and secretion were increased in the CLCs that were prone to degenerate.^23^ However, it is unclear how overexpression of Smurf2 in *Col2a1* promoter‐working CLCs causes the cells to increase CTGF expression and secretion during disc degeneration in *Col2a1‐Smurf2* transgenic mice.

Here, we demonstrated that primary old bovine NP cells and primary young bovine NP cells shared some similarities with the CLCs in transgenic mouse discs and normal cells in wild‐type (WT) mouse discs, respectively. By utilizing this model, we have discovered that TGF‐β induces a rapid increase in β‐catenin, which interacts with CTGF in the cytoplasm and recruits it to the membrane for secretion in old, but not young, NP cells and that when Smurf2 is overexpressed in young NP cells, the cells become senescent and allow the TGF‐β–β‐catenin–CTGF cascade to occur. The TGF‐β‐initiated β‐catenin‐mediated CTGF secretory pathway is novel and is, at least partially, responsible for disc degeneration progression and fibrosis formation.

## Materials and Methods

### Cell isolation and culture

Mouse sternal chondrocytes were isolated as described previously.^22^ Three‐month‐ and 3.5‐year‐old bovine tails were collected immediately after death. Two to three discs were collected from each tail, and assessed as a degenerative grade 2.1 for young discs and 4.1 for old discs based on a scale of 1 to 5,^16^ consistent with previous reports.^14^, ^24^ Four to five tails were used in each experiment. The NP were cut into small pieces, digested with 0.19% Pronase (Roche, Mannheim, Germany) for 1 hour, and was further digested with 0.02% collagenase type II (Worthington Biochemical Corporation, Lakewood, NJ, USA) in DMEM containing 10% FBS o/n, and filtered through a 70‐μm strainer. NP cells were cultured in 6‐cm dishes (1 × 10^6^/dish) or 6‐well plates (0.6 × 10^6^/well) with DMEM supplemented with 10% FBS and 2 mM glutamine.

### Cell infection with lentiviruses and transfection with siRNA

Lentiviruses expressing Smurf2/GFP were assembled by transfecting the 293T cells with pCMVR8.74 (Addgene, Cambridge, MA, USA), pMD2.G VSVG (Addgene), and pLenti‐CMV‐Smurf2/GFP‐IRES‐Puro (provided by Dr H Zhang, University of Massachusetts Medical School) through Fugene 6 (Promega, San Luis Obispo, CA, USA). Media containing lentiviruses was filtered and added to cells. For cell transfection with siRNA, all siRNAs including non‐specific siRNA, siRNA β‐catenin, and siRNA Smurf2 were from Dharmacon (On‐Target Plus; Lafayette, CO, USA) and transfected via Oligofectamine (Invitrogen, Carlsbad, CA, USA).

### Western blot, IP, and ELISA

When cells were 90% confluent, they were treated with MG132 (Calbiochem, MilliporeSigma, Burlington, MA, USA) at 10 µM for 4 hours, chloroquine (Sigma, St. Louis, MO, USA) at 50 μM for 2 to 4 hours, and wortmannin (Sigma) at 200 nM for 30 minutes. After removal of these reagents, TGF‐β1 (Calbiochem) was added at 5 ng/mL. Cytoplasmic lysates were prepared by mechanical shearing of cells suspended in a buffer containing 20 mM Tris‐HCl, 100 mM NaCl, 1 mM EDTA, and 0.2% Triton X‐100. After centrifugation and collection of the supernatant, the remaining pellet was resuspended in a buffer containing 50 mM Tris‐HCl, 50 mM NaCl, and 1% Triton X‐100. After centrifugation, the supernatant was membrane lysates. Nuclear extracts were obtained by breaking open cells to release nuclei with hypotonic buffer and extracting nuclear protein with RIPA buffer. ECM‐binding proteins were extracted as described previously.^25^ Each buffer contained fresh protease/phosphatase inhibitors (Roche). Ten to 30 µg of protein extracts were separated by 10% SDS‐PAGE. Antibodies used for blotting include anti‐CTGF, anti‐Smurf2, anti‐β‐catenin, anti‐GSK‐3β, anti‐pSer9‐GSK‐3β, anti‐pSmad2, anti‐pSmad3, and anti‐p53/p21/Bcl‐2, purchased from Abcam (Cambridge, MA, USA) and Cell Signaling (Danvers, MA, USA), which were used at 1:1500 to 2000 and 1:1000 dilution, respectively, except for BCL‐2 used at 1:500. P16^INK4A^(p16) (Sigma) was diluted at 1:500. Immunoprecipitation (IP) was performed by incubating lysates/media/ECM‐extracts with protein A/G Sepharose beads (Sigma) that had been preconjugated with anti‐β‐catenin mouse and anti‐CTGF rabbit antibodies. Conditioned medium was concentrated to 5× by centrifugal filter Devices (Amicon Ultra‐15, MilliporeSigma), and CTGF content was assessed by ELISA (MyBioSource, San Diego, CA, USA).

### Immunofluorescence and SA‐β‐gal staining

Four/8‐well slides were double‐stained with anti‐β‐catenin mouse (Abcam, 1:100) and anti‐CTGF rabbit antibodies (Abcam, 1:200). The secondary antibodies donkey anti‐mouse Fluorescence 594, and donkey anti‐rabbit Fluorescence 488 (ThermoFisher Scientific, Waltham, MA, USA) were diluted at 1:1000. Six‐well plates were stained for senescence‐associated β‐galactosidase (SA‐β‐gal) using Senescence Cells Histochemical Staining Kit (Sigma). Frozen sections was stained with Senescence Detection Kit (Abcam).

### Real‐time reverse transcription polymerase chain reaction (RT‐PCR)

Total RNA was extracted with RNeasy Plus Mini Kit (Qiagen, Valencia, CA, USA). RNA was reverse transcribed into cDNA using SuperScript IV Reverse Transcriptase (ThermoFisher Scientific). RT‐PCR was performed as previously described^22^ by using SYBR green. The primers for type II collagen are 5′‐ggtgagctatgatccgcctc‐3′ and 5′‐gtcctggttgccggacat‐3′. The primers for aggrecan are 5′‐cccgactgatgcttcaatccc‐3′ and 5′‐cagcttctggtctgttgtggt‐3′.

### Study approval

Mouse spine and human disc waste collection was conducted in accordance with the policies and guidelines proposed in a protocol approved by Institutional Animal Care and Use Committee in Baylor Scott and White Healthcare.

## Results

### Similarities between old bovine NP cells and CLCs in Col2a1‐Smurf2 transgenic mouse discs

**Figure 1:**
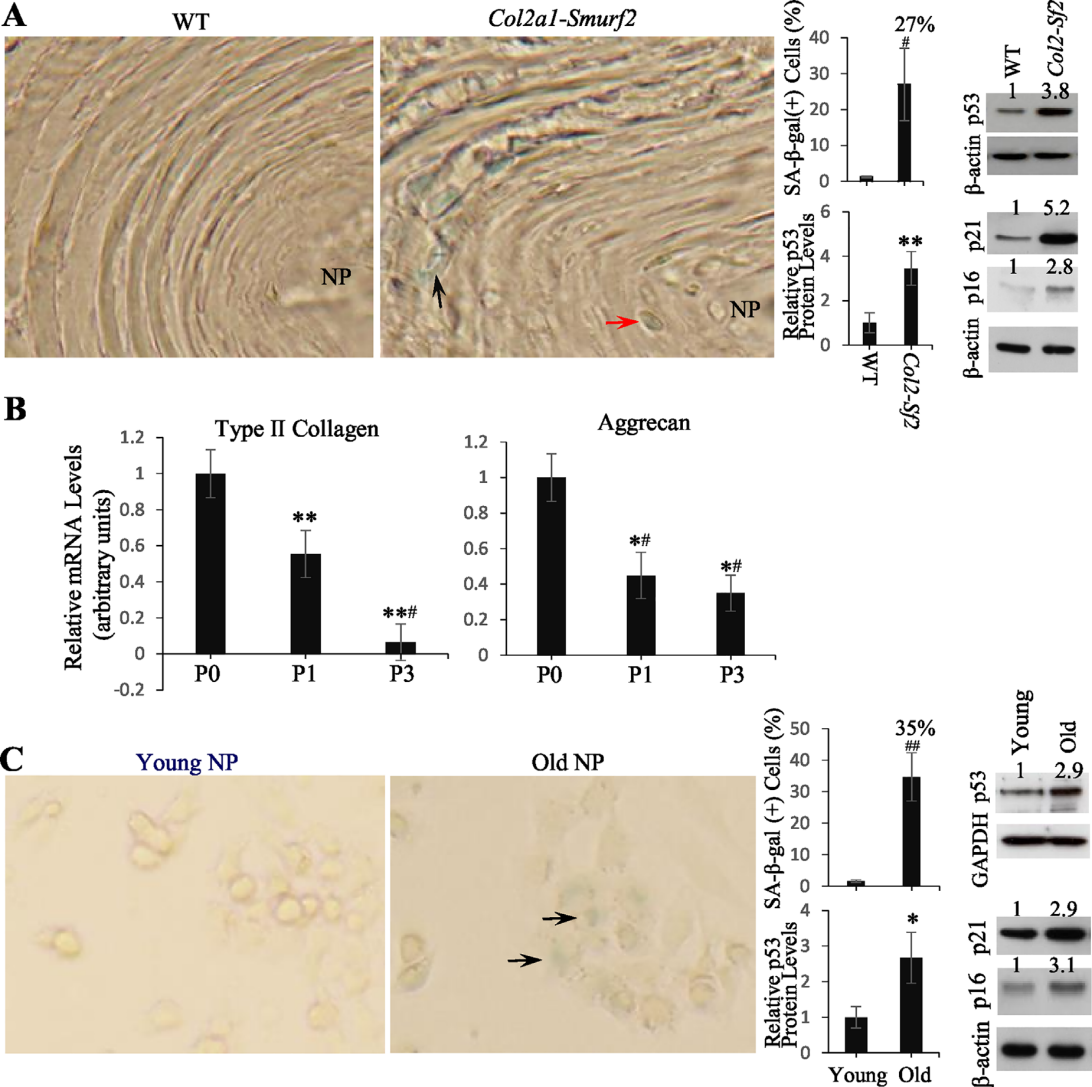
Similarities between old bovine NP cells and chondrocyte‐like disc cells in *Col2a1‐Smurf2* transgenic mice. (*A*) Disc cell senescence in *Col2a1‐Smurf2* transgenic mice. The 5‐μm sagittal frozen sections, prepared from 10‐month‐old mouse lumbar spine, were stained for SA‐β‐gal. Black and red arrows indicate SA‐β‐gal‐(+) cells in the outer AF and inner AF, respectively. Original magnification ×40. Bar graph (upper) is the percentage of SA‐β‐gal‐(+) cells, which was determined by counting all positive and negative cells from 5 fields/section, 2 sections/sample, and a total 3 WT and 3 transgenic samples. Values are the mean ± SEM. Student unpaired *t* test was used. #P = 2.5E‐06. Western blots show an increase in p53, p21, and p16 in transgenic mouse disc cells. Protein extracts for each gel were prepared from disc cells isolated from lumbar discs of 8 WT and 9 transgenic mice without going through cell culture. The values above the blotting bands show results of densitometric analysis of the representative blot (normalized to β‐actin and control was set to 1), which is also true for those in other figures. Bar graph (lower) shows relative p53 protein levels in WT and transgenic disc cells from 3 independent blots. Values are the mean ± SEM. Student unpaired *t* test was used. **p < 0.01. (*B*) Primary old bovine NP cells express type II collagen and aggrecan. The mRNA levels of both genes were decreased as cells were passaged (split ratio is 2), assessed by real‐time RT‐PCR. Values are the mean ± SEM (*n* = 3). Statistical analysis was conducted using 1‐way ANOVA and Student unpaired *t* test. **p < 0.01; *^#^ *p* < 0.005; **^#^ *p* < 0.0001 (versus P0). (*C*) Primary old bovine NP cell senescence. Six‐well plates cultured with primary young and old bovine NP cells were stained for SA‐β‐gal. Arrows indicate SA‐β‐gal‐(+) cells. Original magnification ×40. Bar graph (upper) is the percentage of SA‐β‐gal‐(+) cells, which was determined by randomly choosing 500 cells. Values are the mean ± SEM (*n* = 3). Student unpaired *t* test was used. ^##^P = 5.0E‐07. Western blots show an increase in p53, p21, and p16 in primary old bovine NP cells. Bar graph (lower) shows relative p53 protein levels in young and old bovine NP cells from 3 independent blots. Values are the mean ± SEM. Student unpaired *t* test was used. *p < 0.05.

Because overexpression of Smurf2 in cultured fibroblasts induces senescence^26^ and senescent cell accumulation in discs is important for disc degeneration,^27^ we examined whether the CLCs overexpressing Smurf2 in *Col2a1‐Smurf2* transgenic mouse discs became senescent by examination of senescent markers, SA‐β‐gal, and p53/p21/p16 activation.^26^ It was rare to detect SA‐β‐gal‐positive cells in 10‐month‐old WT mouse discs; however, in the age‐matched transgenic mice, ∼27% of the disc cells, which were mainly CLCs in the AF, were positive (Fig. 1 *A*). Consistently, p53/p21/p16 protein levels in disc cells isolated from 10‐month‐old transgenic mice were higher than that from age‐matched WT mice (Fig. 1 *A*).

Because it is difficult to isolate enough CLCs from *Col2a1‐Smurf2* transgenic mouse discs for further experiments, we wanted to utilize a type of cell that could resemble the CLCs to a certain degree by meeting the following three criteria: 1) a sufficient number of cells can be isolated from fresh disc tissues; 2) they possess chondrocyte phenotype; and 3) a significant percentage of the cells is senescent. Indeed, we could access 3.5‐year‐old and 3‐month‐old young caudal bovine spine from a local abattoir (Waco, TX, USA), and found that primary old bovine NP cells (passage 0) express high levels of type II collagen and aggrecan assessed by the Ct value in RT‐PCR. The mRNA levels of both genes were decreased quickly after cell passages in culture (Fig. 1 *B*), indicating that the primary cells, rather than those that have been passaged, are the most closely related to the chondrocyte phenotype. Approximately 35% of primary old NP cells were SA‐β‐gal‐positive, which contrasted with <2% of primary young NP cells being SA‐β‐gal‐positive (Fig. 1 *C*). In addition, the content of p53/p21/p16 in primary old NP cells was higher than that in primary young NP cells (Fig. 1 *C*). The senescence feather of primary old NP cells was further confirmed by treatment of the cells with dasatinib and quercetin, drugs that target gene products for protecting senescent cells from apoptosis, resulting in downregulation of the senescence‐related genes p21 and p16 as well as an anti‐apoptotic gene Bcl‐2 (Supplemental Fig. S1). It appears that the primary old bovine NP cells are similar to the CLCs in the *Col2a1‐Smurf2* transgenic mouse discs.

### TGF‐β causes a rapid decrease in CTGF protein levels in the cytoplasm of old bovine NP cells

**Figure 2:**
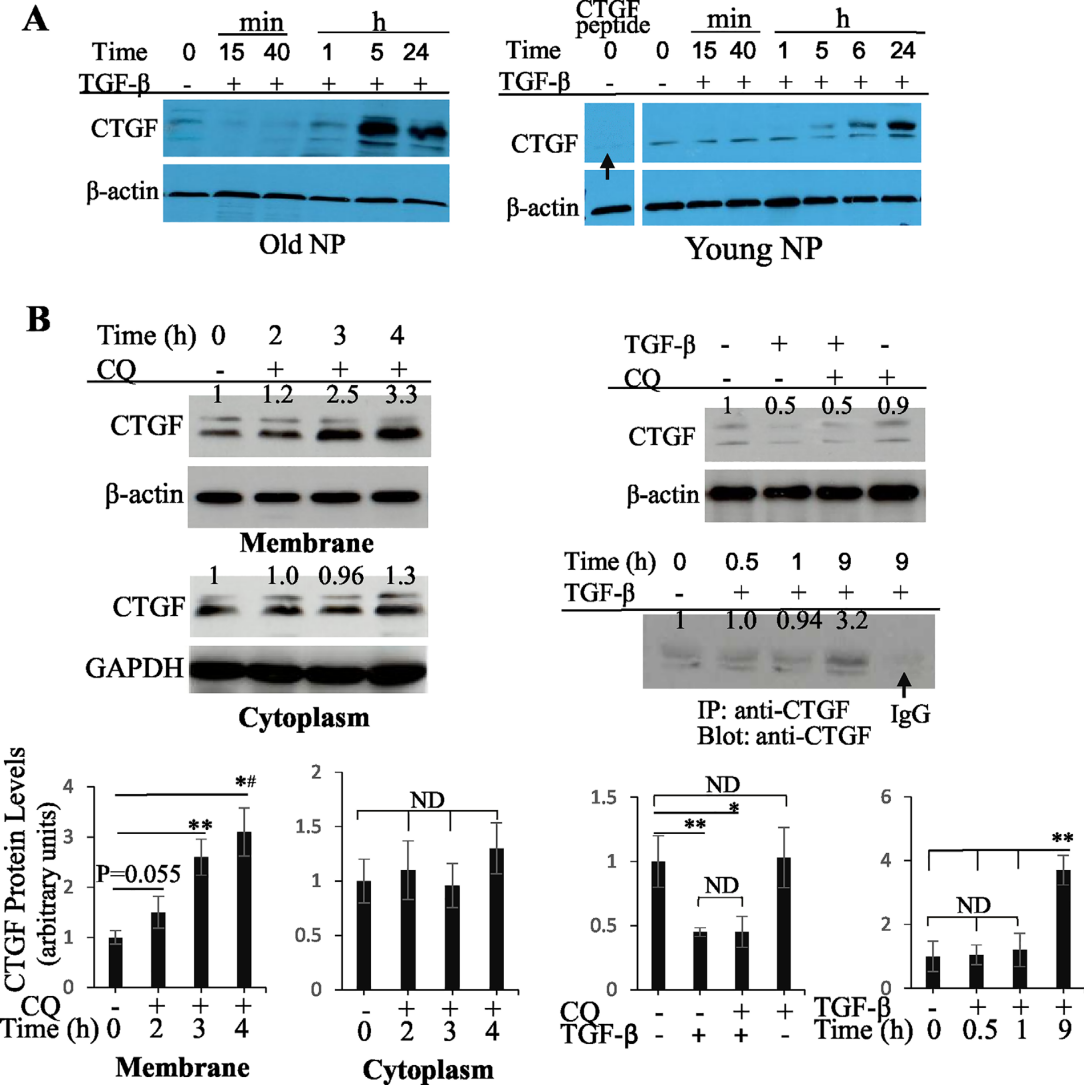
TGF‐β induces a rapid decrease in cytoplasmic CTGF protein levels in old bovine NP cells. (*A*) TGF‐β induces a rapid decrease in cytoplasmic CTGF in old, but not young, bovine NP cells. Cytoplasmic CTGF protein levels were increased by TGF‐β treatment for ≥5 hours in both old and young NP cells; however, they were decreased in the first hour of TGF‐β treatment in old, but not young, NP cells. Arrow indicated that the CTGF bands could not be detected by pre‐incubation of 1 μg anti‐CTGF antibody with 10 μg CTGF peptide for 20 minutes before adding the membrane. (*B*) The rapid decrease in cytoplasmic CTGF in old bovine NP cells is not due to endosomal degradation or secretion. Left panel: inhibition of endosomal degradation pathway does not affect cytoplasmic CTGF protein levels. Old bovine NP cells were treated with chloroquine (CQ) at 50 µM for 2 to 4 hours, and cytoplasmic and membrane lysates were extracted for Western blotting. CTGF in the membrane was significantly increased at 3 hours and 4 hours after CQ treatment; however, CTGF in the cytoplasm at these time points is not changed. Right panel: TGF‐β‐induced rapid decrease in cytoplasmic CTGF is not due to endosomal degradation or secretion. Old bovine NP cells were treated with TGF‐β for 30 minutes with/without pre‐incubation with CQ for 3 hours. TGF‐β induced a decrease in cytoplasmic CTGF, and the decrease could not be rescued by CQ (upper blot). The ECM‐binding CTGF, which was released by incubation of dishes at 85°C for 1 hour with RIPA buffer and immunoprecipitated by anti‐CTGF antibodies, was similar in band density at 0, 0.5, and 1 hour and increased at 9 hours after TGF‐β treatment of cells (lower blot). Arrow indicates the diminished CTGF bands by using normal IgG for precipitation. Bar graphs are quantitative analysis of relative CTGF protein levels in replicates of blots shown above. Values are the mean ± SEM (*n* = 3). Statistical analysis was conducted using 1‐way ANOVA and Student unpaired *t* test. *p < 0.05; **p < 0.01; *^#^ *p* < 0.005; ND = no significant difference.

Because CTGF expression/secretion is increased in CLCs and TGF‐β transcription is increased in disc cells in *Col2a1‐Smurf2* transgenic mice,^(23)^ we want to understand how TGF‐β regulates CTGF protein levels in primary old bovine NP cells. We treated primary old bovine NP cells with TGF‐β and treated primary young bovine NP cells identically as control. Western blot showed that CTGF protein levels were increased in both old and young NP cells after TGF‐β treatment for ≥5 hours (Fig. 2 *A*), consistent with previous studies.^(7)^ However, an unexpected rapid decrease in CTGF protein levels was detected in the cytoplasm of old, but not young, NP cells at 15 and 40 minutes after TGF‐β treatment (Fig. 2 *A*).

To determine whether the rapid decrease in cytoplasmic CTGF in TGF‐β‐treated old NP cells is caused by CTGF secretion, association with ECM, or/and endosomal degradation,^(6,8,10,28)^ we performed three lines of experiments in old NP cells treated with TGF‐β with/without chloroquine, an endosomal inhibitor. First, conditioned media was collected at 30 minutes and 1 hour after TGF‐β treatment, concentrated, and immunoprecipitated with anti‐CTGF antibodies; however, CTGF could not be detected in the concentrated media or the immunoprecipitate via ELISA and Western blot, respectively. Second, chloroquine increased CTGF in the membrane at ≥3 hours after treatment, but cytoplasmic CTGF was not affected (Fig. 2 *B*, left panel). Similarly, the TGF‐β‐initiated rapid decrease in cytoplasmic CTGF could not be rescued by chloroquine (Fig. 2 *B*, right panel). Third, the CTGF bands, corresponding to CTGF peptides dissociated from the ECM, were similar in density at 0, 30 minutes, 1 hour, and 3 hours after TGF‐β treatment; however, the band intensity representing CTGF dissociated from the ECM at 9 hours after TGF‐β treatment was ∼threefold of that in the first 1 to 3 hours (Fig. 2 *B*, right; Supplemental Fig. S2), indicating that CTGF can be retained within the old NP cells after its synthesis for at least 1 hour, consistent with a previous chasing result in chondrocytes.^10^ Thus, the TGF‐β‐induced rapid decrease in cytoplasmic CTGF in old NP cells is not due to secretion or degradation but due to CTGF translocation from the cytoplasm into the membrane or the nuclei within the cells.

### TGF‐β causes a rapid increase in β‐catenin protein levels in old bovine NP cells through inactivation of GSK‐3β

**Figure 3:**
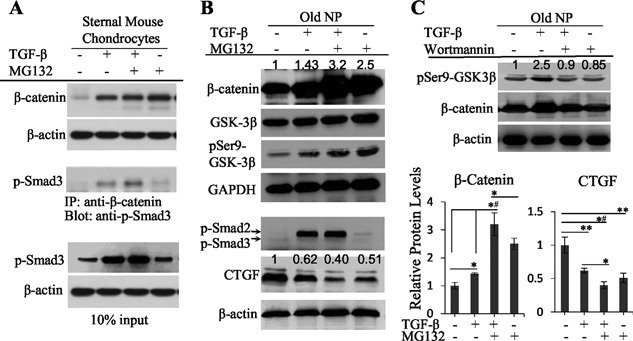
Negative relationship between β‐catenin and CTGF protein levels in the cytoplasm of old bovine NP cells. (*A*) P‐Smad3 interacts with β‐catenin and prevents β‐catenin degradation in proteasome in primary mouse sternal chondrocytes. In mouse sternal chondrocytes, cytoplasmic β‐catenin protein level was dramatically increased by TGF‐β treatment for 30 minutes; it is further increased in the cells treated with MG132, a proteasomal inhibitor (top). The interaction between p‐smad3 and β‐catenin was clearly detected in TGF‐β‐treated cells (middle), which prevented β‐catenin from proteasomal degradation. (*B*) Cytoplasmic β‐catenin and CTGF protein levels were inversely related in old bovine NP cells. In old bovine NP cells, the cytoplasmic protein levels of β‐catenin and pSer9‐GSK‐3β were increased at 30 minutes after TGF‐β treatment, which were further increased by MG132 (top). In contrast to the cytoplasmic β‐catenin pattern, the cytoplasmic CTGF was decreased in the variously treated cells (bottom). The negative relationship between CTGF and β‐catenin protein levels is shown in C (bar graphs). Values are the mean ± SEM (*n* = 3). Statistical analysis was conducted using 1‐way ANOVA and Student unpaired *t* test. *p < 0.05; **p < 0.01; *^#^ *p* < 0.005. P‐Smad2, but not p‐Smad3, was clearly detected in the cytoplasm of TGF‐β‐treated cells (middle). (*C*) TGF‐β‐induced rapid increase in cytoplasmic pSer9‐GSK‐3β and β‐catenin in old bovine NP cells is PI3K dependent. The protein levels of pSer9‐GSK‐3β and β‐catenin were increased in old NP cells by TGF‐β treatment for 30 minutes. The increase of these proteins was abrogated by pre‐incubation of the cells with Wortmannin, a PI3K inhibitor.

Because the TGF‐β‐induced rapid decrease in cytoplasmic CTGF in old NP cells is exactly the opposite of the TGF‐β‐induced rapid increase in β‐catenin in chondrocytes, which is the result of interaction between phosphorylated Smad3 (p‐Smad3) and β‐catenin and protection of β‐catenin from ubiquitination and proteasomal degradation,^29^ we examined whether TGF‐β could cause a rapid increase in β‐catenin protein levels in old NP cells via a similar mechanism. Mouse sternal chondrocytes were used as a positive control. Western blot showed that the basal level of β‐catenin in the cytoplasm of mouse sternal chondrocytes was low; it was dramatically increased by TGF‐β and only slightly further increased by MG132, a proteasomal inhibitor (Fig. 3 *A*, top). P‐Smad3, but not p‐Smad2, interacted with β‐catenin (Fig. 3 *A*, middle), and preserved β‐catenin in TGF‐β‐treated cells at a level similar to that in MG132‐treated cells (Fig. 3 *A*, top).^29^ As compared with the β‐catenin pattern in the cytoplasm of mouse sternal chondrocytes, the basal level of cytoplasmic β‐catenin in old NP cells was high; β‐catenin was moderately increased by TGF‐β and similarly further increased by MG132 (Fig. 3 *B*, top). However, p‐Smad3 was barely detected, although p‐Smad2 was high in TGF‐β‐treated cells (Fig. 3 *B*, middle), indicating that the TGF‐β‐induced rapid increase in cytoplasmic β‐catenin in old NP cells is not achieved via interaction between p‐Smad3 and β‐catenin.

Because the protein levels of GSK‐3β and/or the inactivated form of GSK‐3β, GSK‐3β with serine 9 phosphorylation (pSer9‐GSK‐3β), regulate β‐catenin protein levels in dermal fibroblasts during wound healing,^30^ we examined these two proteins in the cytoplasm of old NP cells. Indeed, compared with untreated cells, TGF‐β increased pSer9‐GSK‐3β at 30 minutes after treatment, whereas total GSK‐3β was not changed (Fig. 3 *B*). With respect to the increase in both GSK‐3β and pSer9‐GSK‐3β caused by MG132, it was due to a blockage of GSK‐3β degradation in proteasome.^31^ Because PI3K has been reported to be involved in TGF‐β‐stimulated cellular functions via activation of the ILK/Akt/GSK‐3β pathway,^32^ we treated old NP cells with wortmannin, a PI3K inhibitor, and found that wortmannin abrogated the TGF‐β‐induced increase in pSer9‐GSK‐3β and β‐catenin (Fig. 3 *C*). Thus, TGF‐β‐induced serine 9 phosphorylation of GSK‐3β in old NP cells is through PI3K‐dependent activation of ILK/Akt.

### β‐Catenin interacts with CTGF in the cytoplasm and recruits it to the membrane of old bovine NP cells

**Figure 4:**
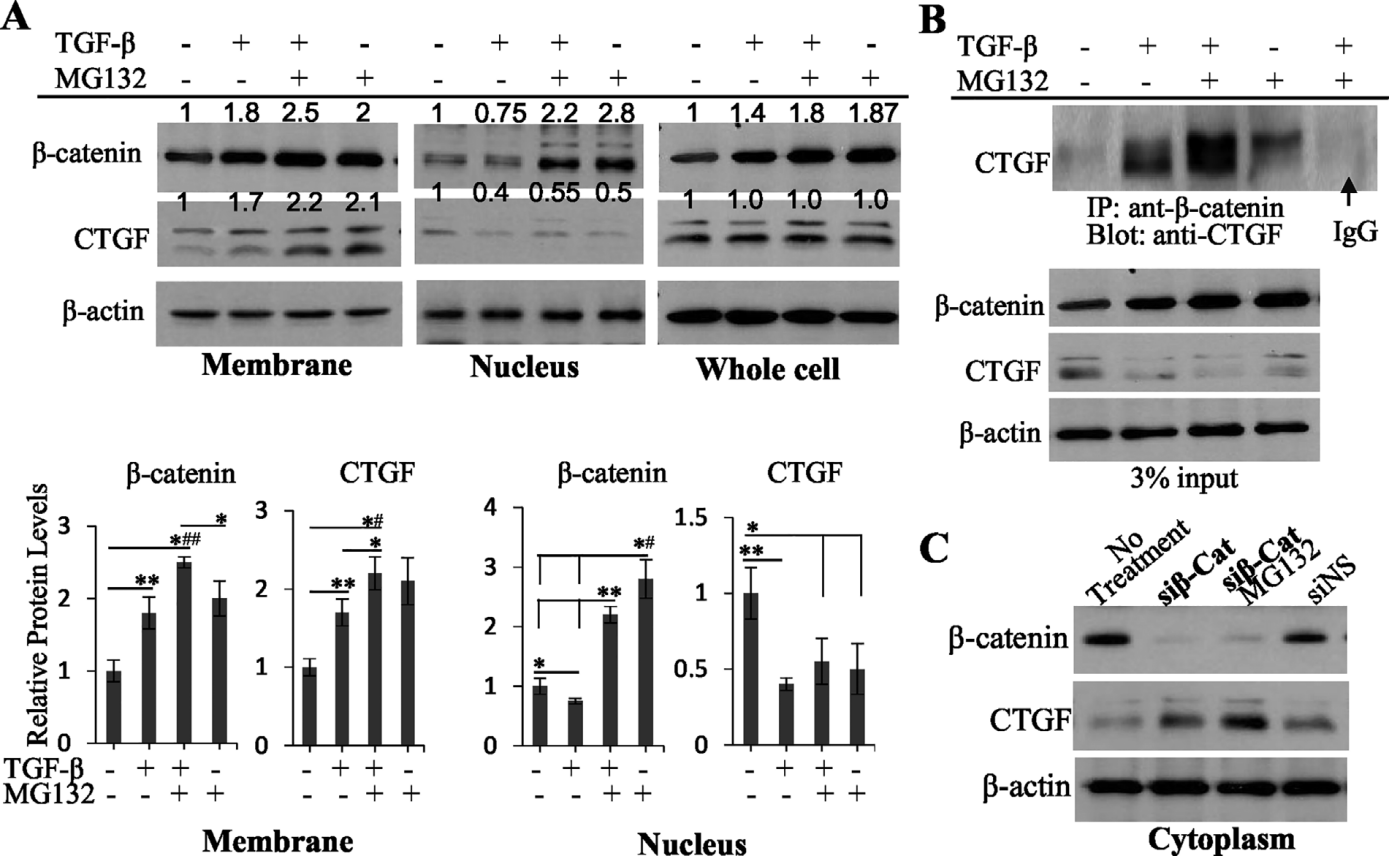
Various relationships between β‐catenin and CTGF in fractionated old bovine NP cells. (*A*) β‐Catenin and CTGF protein levels were positively related in the membrane and negatively related in the nuclei of old bovine NP cells at 30 minutes after TGF‐β treatment. Bar graphs are shown as the mean ± SEM (*n* = 3). Statistical analysis was conducted using 1‐way ANOVA and Student unpaired *t* test. *p < 0.05; **p < 0.01; *^#^ *p* < 0.005; *^##^ *p* < 0.001. (*B*) β‐Catenin interacted with CTGF in the cytoplasm of old bovine NP cells at 30 minutes after TGF‐β treatment. Arrow indicates the diminished CTGF bands by using normal IgG for precipitation. (*C*) Knockdown of β‐catenin causes CTGF accumulation in the cytoplasm of old NP cells. In the cells transfected with siRNA β‐catenin (siβ‐Cat) with/without MG132, CTGF protein levels are higher than those in the cells transfected with non‐specific siRNA (siNS) or the control cells (no treatment).

Because β‐catenin shuttles three pools of the cytoplasm, the nucleus, and the membrane within a cell,^(33)^ we wanted to understand whether the TGF‐β‐induced increase in cytoplasmic β‐catenin in old NP cells could recruit CTGF from the cytoplasm to the membrane or nucleus. First, we examined β‐catenin and CTGF protein levels in fractionated and whole cell lysates via Western blot. In the cytoplasm, β‐catenin and CTGF protein levels were inversely related. For example, β‐catenin was 1.43‐, 3.2‐, and 2.5‐fold increased, whereas CTGF was decreased to 62%, 40%, and 51% in the cells treated with TGF‐β, TGF‐β and MG132, and MG132 only, respectively, versus that in untreated cells (set to 1) (Fig. 3 *B*, bar graphs in 3*C*). In contrast, whereas β‐catenin was increasing gradually as cells were treated variously, CTGF was increased proportionally in the membrane (Fig. 4 *A*). The β‐catenin and CTGF relationship in the nucleus was basically similar to that in the cytoplasm, except that β‐catenin was slightly decreased by TGF‐β (Fig. 4 *A*). Although the β‐catenin pattern in whole cells was similar to that in fractionated cells, the CTGF protein levels were not changed (Fig. 4 *A*). Second, we examined whether β‐catenin interacted with CTGF in the cytoplasm of old NP cells (Fig. 4 *B*). IP revealed that no clear interaction band was detected in untreated cells; it was clearly detected in the cells treated with TGF‐β; it became stronger in the cells treated with TGF‐β and MG132; however, it became weaker in the cells treated with MG132 only, although the β‐catenin protein level in the MG132‐only‐treated cells was similar to that in the cells treated with TGF‐β and MG132 (Fig. 4 *B*). These data indicate that TGF‐β signaling facilitates but is not essential for the interaction between β‐catenin and CTGF in the cytoplasm, which leads to an export of CTGF from the cytoplasm and nucleus into the membrane. Third, knockdown of β‐catenin in old NP cells with siRNA β‐catenin (si‐β‐Cat) caused an accumulation of CTGF in the cytoplasm (Fig. 4 *C*), suggestive of a blockage of CTGF translocation. Therefore, β‐catenin is an essential mediator for CTGF translocation from the cytoplasm into the membrane.

**Figure 5:**
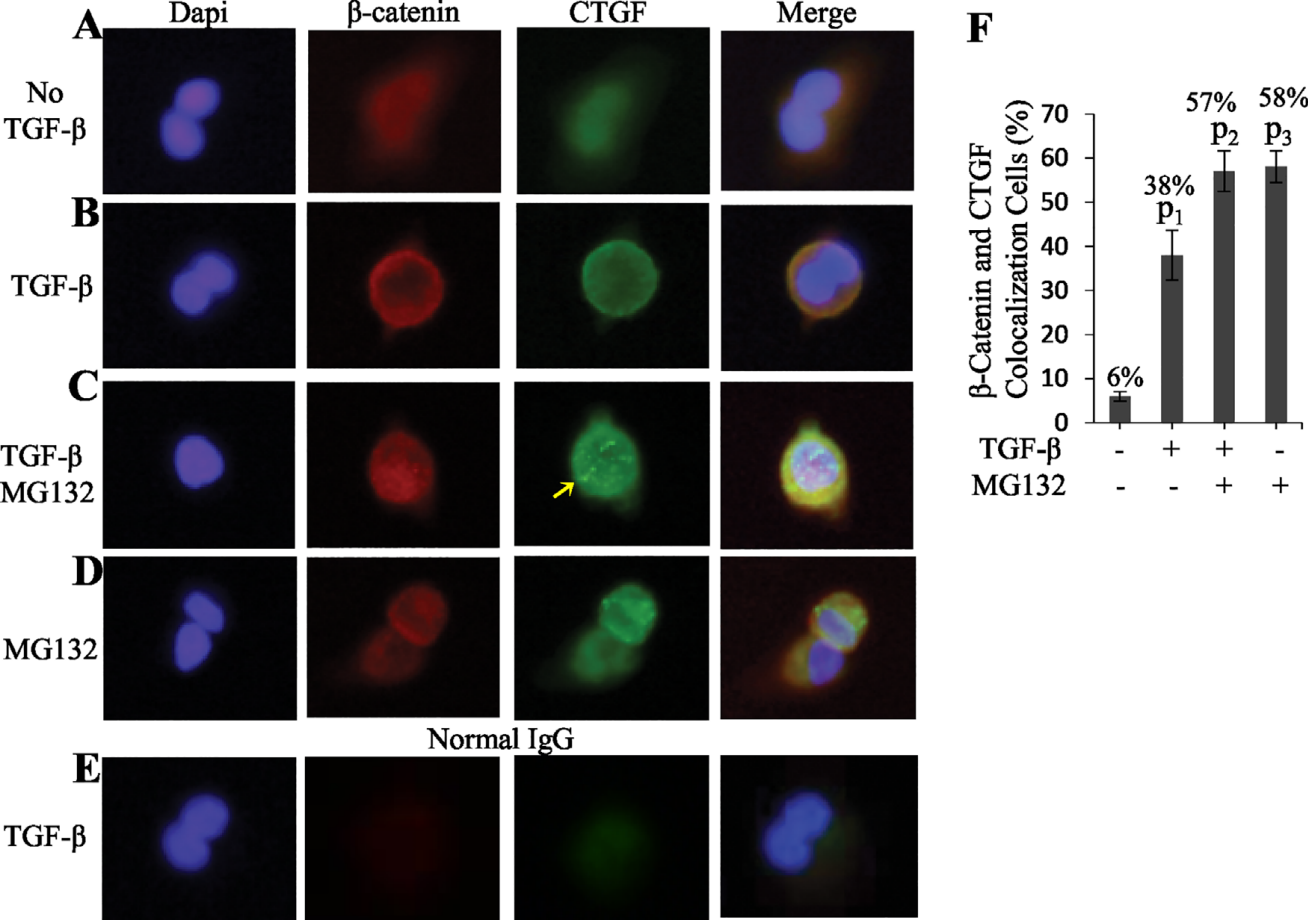
Colocalization of β‐catenin with CTGF in the plasma membrane of old bovine NP cells. (*A*) Endogenous β‐catenin and CTGF are normally distributed in entire old bovine NP cells in a diffuse manner. (*B*) Accumulation and colocalization of β‐catenin and CTGF in the plasma membrane of old bovine NP cells. Cells were treated with TGF‐β for 30 minutes and stained with anti‐β‐catenin and anti‐CTGF antibodies. (*C*, *D*) Increase in β‐catenin and CTGF protein levels and their co‐localization in the plasma membrane of old bovine NP cells. Cells were pretreated with MG132 for 4 hours before 30‐minute TGF‐β treatment. Arrow indicates speckles. (*E*) Background signals in negative controls. β‐Catenin and CTGF were not detected in TGF‐β‐treated old bovine NP cells when normal IgG was used during staining. (*A–E*) Original magnification ×100. (*F*) Quantification of β‐catenin and CTGF colocalization cells. The percentage of colocalization cells was determined from 60 control cells, 90 TGF‐β‐treated cells, 86 TGF‐β + MG132‐treated cells, and 69 MG132‐treated cells from 3 independent experiments. Values are the mean ± SEM. One‐way ANOVA and Student unpaired *t* test were used. p_1_ = 0.0003; p_2_ = 5.5E‐05; p_2_ = 2.7E‐06 (versus control).

The blot and IP results described above were further confirmed by double immunofluorescence staining of old NP cells. As shown in Fig. 5, β‐catenin molecules (red) were normally distributed in whole cells in a diffuse manner (Fig. 5 *A*); in the cells treated with TGF‐β, the amount of β‐catenin was increased in the membrane forming a circumferential ring (Fig. 5 *B*). In the cells treated with TGF‐β and MG132 and MG132 only, in addition to the circumferential ring, β‐catenin was also increased in the nucleus (Fig. 5 *C*, *D*). Like β‐catenin in control cells, CTGF (green) was distributed evenly in whole cells (Fig. 5 *A*). In TGF‐β‐treated cells, CTGF was increased in the membrane and colocalized with β‐catenin at the cell periphery (Fig. 5 *B*). In the cells treated with TGF‐β and MG132, and MG132 only, CTGF in the membrane was further increased versus TGF‐β‐treated cells and colocalized with β‐catenin (Fig. 5 *C*, *D*). In addition, green speckles were frequently observed in the cytoplasm or near the nucleus (Fig. 5 *C*, arrow), which were presumably representing CTGF localized in the ER, the Golgi, or in the vesicles on their way to the membrane.^28^ Statistical analysis revealed that 38%, 57%, and 58% of old NP cells respectively treated with TGF‐β, TGF‐β and MG132, and MG132 only showed colocalization of β‐catenin and CTGF in the membrane, whereas only 6% of untreated cells showed similar colocalization (Fig. 5 *F*). The TGF‐β‐induced β‐catenin and CTGF colocalization found in old bovine NP cells was also detected in disc cells from *Col2a1‐Smurf2* transgenic mice. It was rare to detect β‐catenin and CTGF colocalization in TGF‐β‐treated WT disc cells; however, ∼32% of TGF‐β‐treated transgenic disc cells exhibited β‐catenin and CTGF colocalization (Supplemental Fig. S3).

### Young bovine NP cells become senescent and obtain characteristics of old bovine NP cells after gain of Smurf2 function

**Figure 6:**
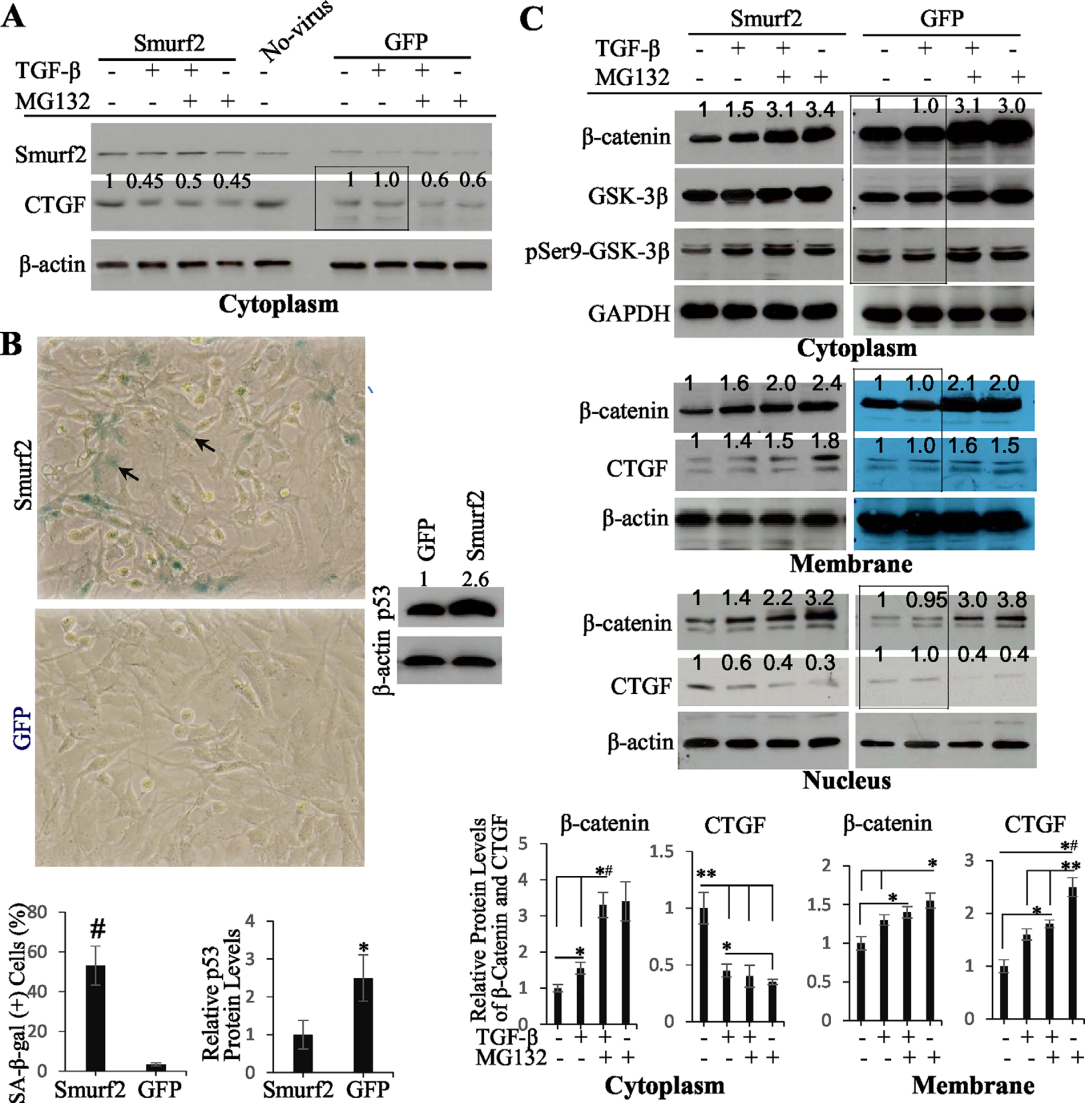
Young bovine NP cells become senescent and obtain characteristics of old bovine NP cells after gain of Smurf2 function. (*A*) A rapid decrease in cytoplasmic CTGF protein levels in young bovine NP cells after gain of Smurf2 function. Young NP cells were incubated with Smurf2/GFP‐expressing lentiviruses for 3 days and treated variously. Western blot showed that Smurf2 protein levels were higher in Smurf2‐infected cells than those in GFP‐infected or no‐virus‐infected cells. Cytoplasmic CTGF protein levels were decreased at 30 minutes after TGF‐β treatment in Smurf2‐infected, but not GFP‐infected (boxed area), cells. (*B*) Young bovine NP cells become senescent after gain of Smurf2 function. SA‐β‐gal‐(+) cells are shown in Smurf2‐infected, but not GFP‐infected, cells (arrows). Original magnification ×40. Bar graph (left) is the percentage of SA‐β‐gal‐(+) cells. Statistical analysis was performed as described in Fig. 1 *C*. #P = 6.4E‐07. Western blot showed that the protein level of p53 in Smurf2‐infected cells was higher than that in GFP‐infected identical cells. Bar graph (right) is shown as the mean ± SEM (*n* = 3 blots). Student unpaired *t* test was used. *p < 0.05. (*C*) Various relationships between β‐catenin and CTGF protein levels in fractionated young bovine NP cells after gain of Smurf2 function. A rapid increase in cytoplasmic β‐catenin and pSer9‐GSK‐3β protein levels was detected at 30 minutes after TGF‐β treatment in Smurf2‐infected, but not GFP‐infected (boxed area), cells (top). The cytoplasmic β‐catenin and cytoplasmic CTGF protein levels (shown in *A*) were negatively related (bar graphs, cytoplasm). The β‐catenin and CTGF protein levels were positively related in the membrane of Smurf2‐infected, but not GFP‐infected (boxed area), cells (middle) (bar graphs, membrane). Bar graphs are presented as the mean ± SEM (*n* = 3 blots). One‐way ANOVA and Student unpaired *t* test were used. *p < 0.05; **p < 0.01; *^#^ *p* < 0.005. The β‐catenin and CTGF protein levels were negatively related in the nuclei of Smurf2‐infected, but not GFP‐infected (boxed area), cells (bottom).

Because TGF‐β induced a rapid decrease in cytoplasmic CTGF in old but not young NP cells, we wanted to determine whether overexpression of Smurf2 in young NP cells would induce senescence and allow TGF‐β to initiate a β‐catenin‐mediated CTGF recruitment cascade. We first overexpressed Smurf2 in young NP cells by infecting the cells with the lentivirus‐based vector pLenti‐CMV‐Smurf2‐IRES‐Puro and an identical construct expressing GFP as control.^(26)^ We found that Smurf2 protein levels in Smurf2‐infected cells were two‐ to threefold of that in GFP‐infected or no‐virus‐infected cells (Fig. 6 *A*); ∼50% of Smurf2‐infected cells and 4% of GFP‐infected cells were SA‐β‐gal‐positive (Fig. 6 *B*); p53 protein level in Smurf2‐infected cells was 2.6‐fold of that in GFP‐infected cells (Fig. 6 *B*). We secondly treated the cells variously and examined CTGF and β‐catenin protein levels in fractionated cell lysates. As found in old NP cells, CTGF protein levels in the cytoplasm of Smurf2‐infected young NP cells were decreased at 30 minutes after TGF‐β treatment with/without MG132 (Fig. 6 *A*), which was accompanied by a simultaneous increase in β‐catenin and pSer9‐GSK‐3β (Fig. 6 *C*); in the membrane and nucleus, a positive and negative relationship between β‐catenin and CTGF protein levels was detected, respectively (Fig. 6 *C*). In the GFP‐infected cells, however, the protein levels of CTGF, β‐catenin, and pSer9‐GSK‐3β were not changed by TGF‐β (Fig. 6 *A*, *C*, boxed areas) in spite of a negative CTGF–β‐catenin relationship in the cytoplasm and a positive relationship in the membrane in the presence of MG132 (Fig. 6 *A*, *C*). These results indicate that when Smurf2 is overexpressed in young NP cells, the cells become senescent and prime a cellular condition for TGF‐β to activate pSer9‐GSK‐3β–β‐catenin pathway and hence to initiate a β‐catenin‐mediated CTGF translocation.

### TGF‐β loses its ability to activate pSer9‐GSK‐3β–β‐catenin–CTGF translocation cascade in old bovine cells after loss of Smurf2 function

**Figure 7:**
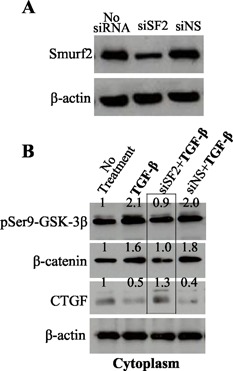
TGF‐β‐initiated CTGF translocation is blocked in old NP cells after loss of Smurf2 function. (*A*) Knockdown of Smurf2 in old bovine NP cells using siRNA Smurf2 (siSF2). (*B*) CTGF accumulation in the cytoplasm of old NP cells after loss of Smruf2 function. pSer9‐GSK‐3β and β‐catenin were increased and CTGF was decreased in the cells treated with TGF‐β for 30 minutes with/without non‐specific siRNA (siNS) transfection versus no treatment; however, TGF‐β failed to do so in the cells transfected with siRNA Smurf2 (boxed area).

Given that senescence transition is highly dynamic,^(34)^ knockdown of Smurf2 in old bovine NP cells could cause loss of the ability of TGF‐β to activate GSK‐3β–β‐catenin pathway and subsequent CTGF translocation. To test this, we knocked down Smurf2 protein level by ∼70% in old bovine NP cells using siRNA Smurf2 (siSF2) (Fig. 7 *A*), and treated the cells with TGF‐β for 30 minutes. We found that in the cells transfected with non‐specific siRNA (siNS), TGF‐β increased pSer9‐GSK‐3β and β‐catenin and decreased CTGF protein levels in the cytoplasm versus those in no‐treatment control (Fig. 7 *B*), similar to the changes in TGF‐β‐treated cells (Fig. 7 *B*); however, in the TGF‐β‐treated cells transfected with siSF2, pSer9‐GSK‐3β and β‐catenin were not increased, which led to CTGF accumulation in the cytoplasm (Fig. 7 *B*, boxed area). This data implies that loss of Smurf2 within old bovine NP cells reverses cell stage toward young direction.

## Discussion

Although disc degenerative diseases are globally prevalent and impose high economic burdens, the precise molecular cascades that control disc degeneration process are unknown.^13^, ^35^ Disc degeneration is age related and affected by many risk factors, such as excessive mechanical loading, obesity, trauma, nutrition, smoking, and inflammation, as well as catabolic cytokines and proteases, all of which can weaken discs and lead to the acceleration of the disc degeneration process;^13^, ^17^ however, none of these can explain the disc degeneration process properly. Senescent cell accumulation in discs plays a central role in disc degeneration because all the factors are either senescence‐inducing stresses or a consequence of senescent cells.^35^ Senescent cells cease proliferation but remain metabolically active. Senescent cell numbers in discs are correlated with disc degeneration severity, which is because senescent cells not only reduce the ability to repair but also secrete matrix degradation enzymes leading to expansion of structural defects.^27^, ^35^ However, these findings still cannot account for why moderate disc degenerate phenotypic changes such as cell cloning, migration, and matrix deposition adjacent to structural defects will eventually progress into fibrotic and scar‐like tissue of entire discs. Smurf2 was originally found to be an E3 ubiquitin ligase, which targets the TGF‐β receptor and Smads for ubiquitination and proteasomal degradation.^36^, ^37^ However, a senescence‐inducing activity of Smurf2 was reported in cultured fibroblasts, which was independent of its ubiquitin ligase activity.^(26)^ Consistent with its senescence‐inducing activity in fibroblasts, the disc cells that express type II collagen and Smurf2, driven by the *Col2a1* promoter, are senescent in *Col2a1‐Smurf2* transgenic mice (Fig. 1). These cells are chondrocyte‐like, located in the AF, and are prone to degenerate as disc degeneration progresses.^23^ Because of the difficulty of obtaining enough CLCs from *Col2a1‐Smurf2* transgenic discs, we employed primary old bovine NP cells that express chondrocyte‐specific matrix proteins and possess a senescent phenotype as substitutes for the CLCs to study how these cells contribute to the disc degeneration progression in *Col2a1‐Smurf2* transgenic mice. Given that the phenotypic changes that occurred during disc degeneration and progression in *Col2a1‐Smurf2* transgenic mice could reflect CTGF functions during wound healing and scleroderma, we hypothesized that incomplete repair of clefts/tears in *Col2a1‐Smurf2* transgenic mouse discs caused a persistence of CTGF expression and secretion by the CLCs adjacent to the structural defects due to continuous production and release of TGF‐β by these cells as a cellular response to repetitive excessive deformation of disrupted matrix.^17^, ^38^, ^39^ We tested this hypothesis by examining how TGF‐β regulated CTGF protein levels in old bovine NP cells. As found in other types of cells, TGF‐β increased CTGF protein levels in the cytoplasm of old NP cells at ≥5 hours after TGF‐β treatment, consistent with a lack of requirement of de novo protein synthesis for an immediate early gene expression.^7^ However, an unexpected rapid decrease in cytoplasmic CTGF protein level at 30 minutes after TGF‐β treatment was observed. As the TGF‐β‐induced rapid decrease in cytoplasmic CTGF in old NP cells was exactly the opposite of the TGF‐β‐induced rapid increase in β‐catenin in chondrocytes,^29^ we examined β‐catenin in TGF‐β‐treated old NP cells. Although TGF‐β induced a rapid increase in cytoplasmic β‐catenin in old NP cells, the degree of increase was moderate. Moreover, the increased β‐catenin did not translocate into the nucleus but rather moved to the membrane, consistent with a previous report that a threshold level of β‐catenin accumulation was required for its nuclear translocation.^33^ These data indicate distinct mechanisms regulating β‐catenin accumulation in the cytoplasm of old NP cells and chondrocytes and demonstrates a molecular basis for the cytoplasmic β‐catenin driving cytoplasmic CTGF to the membrane for secretion in old NP cells.

By utilizing blotting, IP, and immunofluorescence, we found that TGF‐β‐induced accumulation of β‐catenin in the cytoplasm of old NP cells interacted with CTGF and recruited it to the membrane for secretion (Figs.  3–5). By contrast, the TGF‐β‐initiated β‐catenin‐mediated CTGF translocation cascade could not be detected in young NP cells (Figs. 2 and 6). Importantly, this cascade was allowed to occur in young NP cells after the cells became senescent via gain of Smurf2 function (Fig. 6). Because senescence transition is highly dynamic,^34^ disc cells are heterogeneous, and CLCs are at various states in vivo, the disc degeneration phenotype in *Col2a1‐Smurf2* transgenic mice could be a result of coordination of all these biological features. Therefore, this mechanism can explain how overexpression of Smurf2 in CLCs accelerates disc degeneration in *Col2a1‐Smurf2* transgenic mice to a certain degree. First, when Smurf2 was overexpressed in the inner AF cells that express type II collagen during disc development, the cells started a senescence transition, reducing the ability to proliferate and synthesize matrix and leading to a decrease in cartilage matrix content at 2 months of age.^23^ Because matrix architecture in the inner AF was altered, moderate/severe clefts and tears occurred at 6 months of age.^23^ Second, the cells adjacent to the disrupted structure produced and released TGF‐β, which promoted CTGF production and secretion by local cells. Secreted CTGF, in turn, attracted fibroblasts in the outer AF to the disrupted area for repair.^23^ In addition, secreted CTGF in a local microenvironment could directly bind TGF‐β and enhance TGF‐β binding to its receptors;^40^ therefore, a high level of CTGF could be maintained even in the presence of a low level of TGF‐β. Third, because the structural defect in discs was never healed, the repairing process was not terminated and a high CTGF protein level persisted, leading to extra scarring and fibrosis. Lastly, the TGF‐β‐induced activation of the CTGF secretory pathway in Smurf2‐expressing disc cells, a potential mechanism for disc degeneration in *Col2a1‐Smurf2* transgenic mice, could also be a mechanism for disc aging/degeneration in WT mice and humans. This could occur because endogenous TGF‐β, produced and released by the cells adjacent to disrupted disc structure,^17^, ^38^, ^39^ could stimulate expression of Smurf2 by these cells and their neighboring cells in an autocrine and paracrine manner, a process formulated based on our findings that TGF‐β stimulated Smurf2 gene expression in chicken chondrocytes^41^ and bovine disc cells (Supplemental Fig. S4); the intensity of Smurf2‐immunostaining‐positive cells adjacent to structural defects of old bovine discs was much stronger than that in relatively normal regions (Supplemental Fig. S5); and Smurf2 was highly detected in human degenerated discs but not in normal controls (Supplemental Fig. S6). Although endogenous TGF‐β most likely initiates the CTGF secretory pathway in Smurf2‐expressing cells in aging/degenerate discs in a focal manner, secreted CTGF functions as a matricellular protein and eventually leads to entire disc fibrogenesis.

## Disclosures

All authors state that they have no conflicts of interest.

## Supporting information

Supporting Figures S1.

Supporting Figures S2.
