# Supplementary figures and images for: TGF‐β Initiates β‐Catenin‐Mediated CTGF Secretory Pathway in Old Bovine Nucleus Pulposus Cells: A Potential Mechanism for Intervertebral Disc Degeneration
Source: JBMR Plus. 2018 Jul 10;3(2):e10069. doi: 10.1002/jbm4.10069 (PMC6383704; doi:10.1002/jbm4.10069)

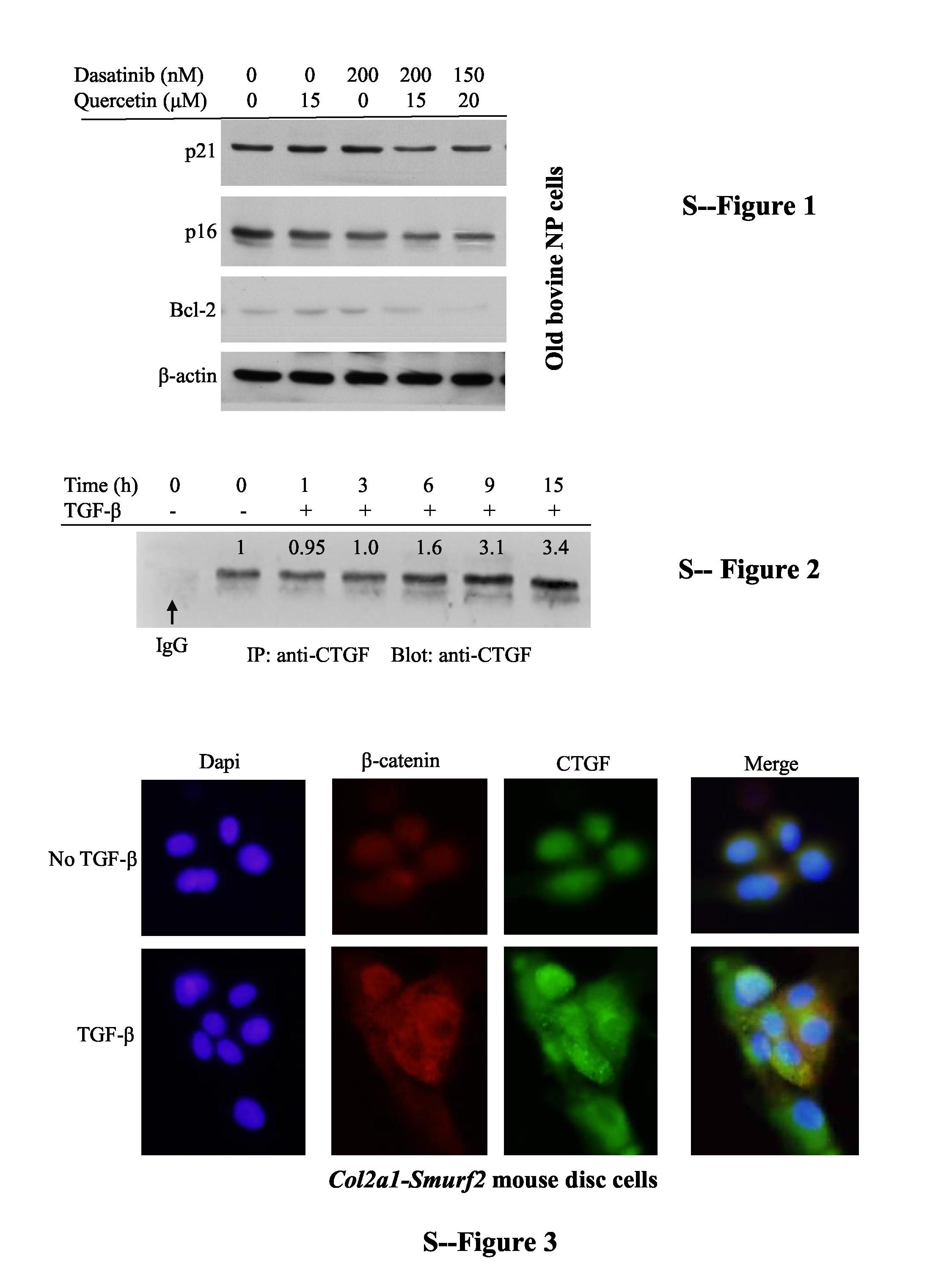

Supplement: Supplementary file 1 — Supporting Figures S1. [file JBM4-3-na-s001.tiff]

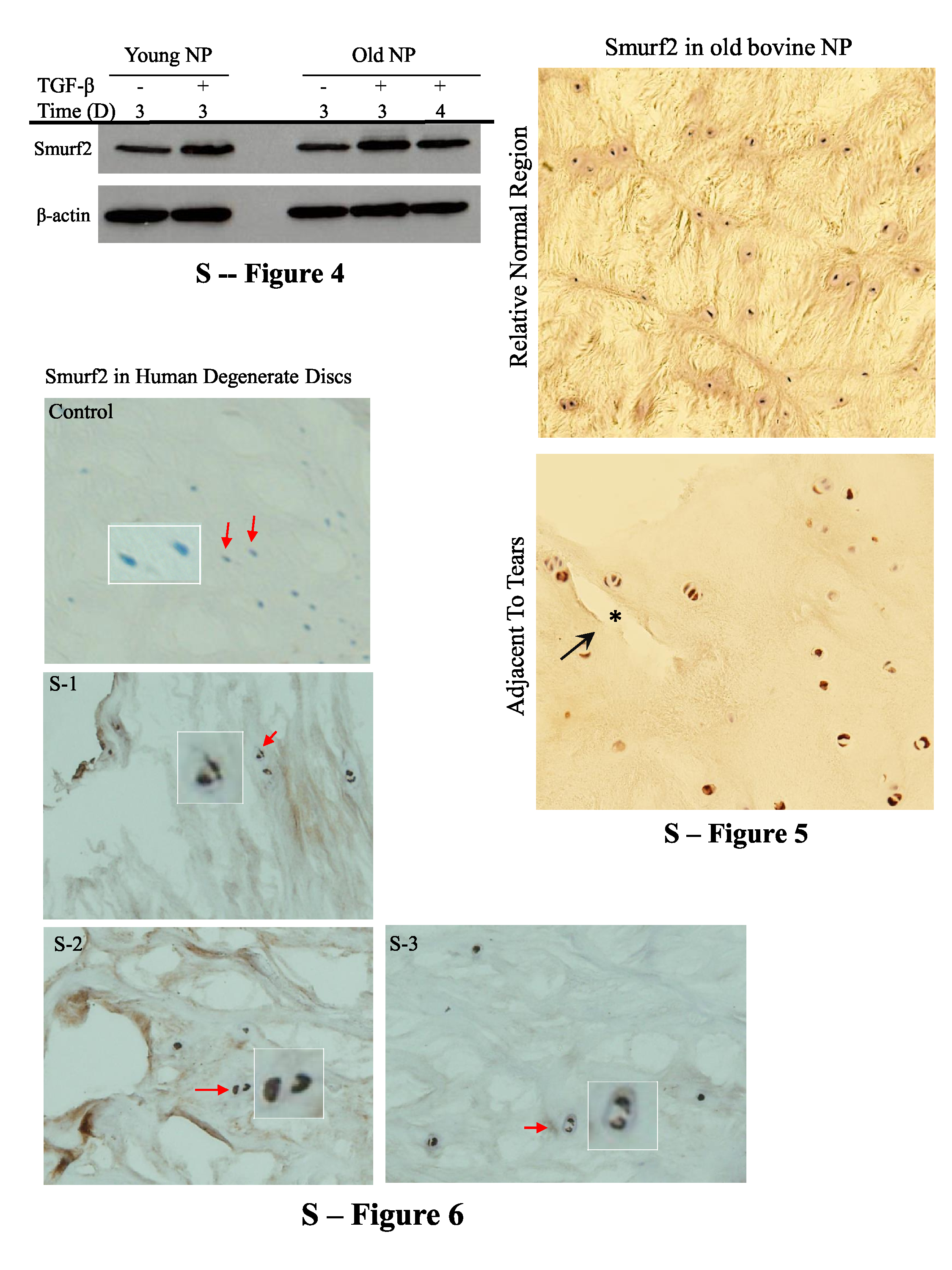

Supplement: Supplementary file 2 — Supporting Figures S2. [file JBM4-3-na-s002.tiff]
